# Arrestin-Coding Genes Regulate Endocytosis, Sporulation, Pathogenicity, and Stress Resistance in *Arthrobotrys oligospora*

**DOI:** 10.3389/fcimb.2022.754333

**Published:** 2022-02-16

**Authors:** Liang Zhou, Mengfei Li, Peijie Cui, Mengqing Tian, Ya Xu, Xi Zheng, Keqin Zhang, Guohong Li, Xin Wang

**Affiliations:** ^1^ State Key Laboratory for Conservation and Utilization of Bio-Resources in Yunnan, Yunnan University, Kunming, China; ^2^ Key Laboratory for Microbial Resources of the Ministry of Education, Yunnan University, Kunming, China

**Keywords:** arrestin, *Arthrobotrys oligospora*, trap formation, sporulation, pathogenicity, pH signal, stress resistance

## Abstract

Arrestins are a family of scaffold proteins that play a crucial role in regulating numerous cellular processes, such as GPCR signaling. The *Arthrobotrys oligospora* arrestin family contains 12 members, which have highly conserved N-terminal and C-terminal domains. In the presence of ammonia, *A. oligospora* can change its lifestyle from saprotrophic to carnivorous. During this transition, the expression pattern of arrestin-coding (*AoArc*) genes was markedly upregulated. Therefore, we disrupted seven *AoArc* genes from *A. oligospora* to identify their functions. Although individual arrestin mutant strains display similar pathogenesis, phenotypes, and stress resistance, the fundamental data on the roles of *AoArc* genes in *A. oligospora* are obtained in this study. Membrane endocytosis in *AoArc* mutants was significantly reduced. Meanwhile, the capacity of trap device formation against nematodes and ammonia was impaired due to *AoArc* deletions. We also found that *AoArc* genes could regulate conidial phenotypes, cell nuclear distribution, pH response, and stress resistance. Results of qRT-PCR assays revealed that sporulation-regulated genes were affected after the deletion of *AoArc* genes. In particular, among the 12 arrestins, *AoArc2* mediates pH signaling in the fungus *A. oligospora*. Notably, combined with the classical paradigm of arrestin–GPCR signal transduction, we suggest that arrestin-regulated trap formation in *A. oligospora* may be directly linked to the receptor endocytosis pathway.

## Introduction

All eukaryotic organisms fine-tune the abundance and activity of cell surface proteins in response to fluctuations in nutrient availability (Boeckstaens et al., 2015). Effective and rapid turnover of membrane transporters is vital for the proper uptake of external cues and signal transduction (Busto et al., 2018). During this process, the endocytosis machinery tightly regulates the stability and dynamics of plasma membrane (PM) proteins, especially G-protein-coupled receptors (GPCR) (Wolfe and Trejo, 2007; Apaja and Lukacs, 2014). GPCRs are integral players in the signal transduction of various biological phenomena. They can respond to and transduce diverse extracellular signals *via* receptor internalization and trigger a series of signaling cascades (Latorraca et al., 2018). Internalizing GPCRs involve a sequential binding of β-arrestin, the clathrin adaptor AP-2, and clathrin, then engage in various signaling activities, such as mitogen-activated protein kinase (MAPK) signaling (Pierce et al., 2000; Laporte et al., 2002).

The proteins of the arrestin family act as adaptor proteins important for the trafficking of nutrients and signaling receptors, especially in the desensitization and endocytosis of transmembrane proteins (Shenoy and Lefkowitz, 2011). Specifically, upon stimulation of an external agonist, β-arrestin is recruited to many activated GPCRs or other membrane receptors. Its subsequent inactivation and internalization lead to receptor degradation and G-protein signal termination. Thus, arrestin functions as a desensitizing molecule that is an important regulatory aspect of the receptor endocytosis process (Jean-Charles et al., 2017). The arrestin family constitutes the highly conserved β-class arrestins (visual and β-arrestins) and the α-class arrestins (Aubry et al., 2009). The common feature in the structures of all arrestins is that they contain N-terminal/C-terminal arrestin domains, which contribute to the uncoupling of the receptors downstream of the G-protein after the receptors are phosphorylated by GPCR kinases (Ferguson, 2001).

Among them, α-arrestins are believed as the predecessor of visual and non-visual β-arrestins. Apart from receptor desensitization and internalization like other arrestins, fungal α-arrestins often function in signaling cascades and also act as adaptors facilitating the proper localization of other proteins (Telzrow et al., 2019). Moreover, a general amino acid permease (Gap1) and an arginine-specific permease (Can1) serve as linkers for ubiquitin ligases to facilitate the function of arrestins (Ghaddar et al., 2014): that is, arrestins can bind the WW domains in E3 ubiquitination ligase Rsp5 through prolinerich ubiquitin ligase binding motifs (PxY sites). Subsequently, the receptor proteins are ubiquitinated and endocytosed for further downregulation, thus allowing cells to adapt better to nutrient inavailability and stress stimulation (Baile et al., 2019). In *Saccharomyces cerevisiae*, the ART–Rsp5 ubiquitin ligase network forms a plasma membrane quality control system that can protect yeast cells from proteotoxic stress through endocytosis and degradation of misfolded proteins (Zhao et al., 2013).

Plant-parasitic nematodes cause a dramatic crop loss throughout the world annually (Ahmad et al., 2021). Nematode-trapping fungi (NTF) are a large and diverse group of natural enemies against nematode pathogens in the soil. They can be a powerful weapon in eliminating nematode hazards (Hsueh et al., 2013). The saprotrophic NTF can quickly respond and adapt to environmental changes by carnivorous growth. Such morphological conversion is characterized by the formation of a variety of fungal capturing devices, including adhesive nets, adhesive knobs, adhesive branches, and constricting and non-constricting rings (Zhang et al., 2020). In the presence of nematodes, the predacious fungi enable these special structures to capture and consume nematode pests. Therefore, understanding the mechanism of growth-form conversion in NTF is important for their bio-utilization against nematodes. *Arthrobotrys oligospora* is the most typical NTF and has been used as the model for studying the evolution of trapping devices and the interaction between fungi and nematodes (Ji et al., 2020). This fungal species mainly grows as saprotrophs under nutrient-rich conditions and as predator in the presence of nematodes and other external substrates, such as amino acids, urea, and ascarosides (Friman et al., 1985; Hsueh et al., 2013; Wang et al., 2014). The trapping structure helps *A. oligospora* to immobilize and kill nematodes. To date, many fungal pathogenicity-related genes have been defined, such as the coding genes of mitogen-activated protein kinase Slt2 and the Rab GTPase AoRab-7A (Yang et al., 2018; Zhen et al., 2018).

We previously identified a novel mechanism for maintaining the stability of bacteria, fungi, and nematodes in the soil environment, by which bacteria can induce NTF to capture and kill nematodes in their habitats. Bacteria, facing their predators, can rapidly excrete urea to trigger NTF to form massive adhesive nets to control the nematode population. After the uptake of urea, it can be carbonated with ammonia and CO_2_. Finally, ammonia signals NTF to switch from vegetative to predacious growth (Wang et al., 2014). Therefore, in this study, we used ammonia as an induction cue for trap formation to clarify the roles of arrestin proteins in the growth-form conversion of *A. oligospora*. We documented the functions of arrestins relative to endocytosis, virulence-associated cell differentiation and conidiogenous cell development, and multistress resistance in the nematode-trapping fungus *A. oligospora*.

## Materials and Methods

### Fungal Strains and Culture Conditions

*Arthrobotrys oligospora* Fres. (ATCC 24927) was maintained on a common fungal nutrient medium (potato dextrose agar, PDA) at 28°C, and *AoArc* mutants were cultured on a PDA supplement with hygromycin resistance at 28°C. TGA (1% tryptone, 1% glucose, 1.5% agar) and TYGA (TGA with 0.5% yeast extracts and 1% molasses) were used for fungal phenotype tests and CMYA (CMA with 0.5% yeast extracts) was used for sporulating culture (Long et al., 2021). All the above fungal strains were stored in the culture collection of the Key Laboratory for Conservation and Utilization of Bio-resource, Yunnan University, China. The nematode *Caenorhabditis elegans* N2 was maintained on nematode growth media (NGM) at 20°C (Roh et al., 2010).

### Sequence and Phylogenetic Analyses

All arrestin protein sequences were retrieved from NCBI (ATCC 24927) and UniProt using the search keywords “arrestin and *Arthrobotrys oligospora*”. The individual arrestin homologs from other organisms were searched and downloaded from GenBank. The sequences of arrestins from different organisms were used to construct neighbor-joining trees for arrestins using the MEGA6 software package (Kumar et al., 2016). Conserved arrestin domains were predicted based on Pfam (http://pfam.xfam.org) and SMART (http://smart.embl.de/).

### FM4-64 Staining Analysis

To test endocytosis, the fungus blocks of the wild-type (WT) and mutant strains were cultured on two-layer water agar (WA) plates for 3 days; then, the upper medium was cut and these fungal blocks were stained with 100-fold diluted FM4-64 (Biotium, CA, USA) for 1 and 10 min, respectively. Then, the samples were washed with ddH_2_O three times and observed with a fluorescence microscope (Nikon, Tokyo, Japan) (Ma et al., 2020).

### Deletion of *AoArc* Genes

The sequences of seven *AoArc* genes were downloaded from the whole genome sequence on the NCBI website. Gene knockout was carried out according to the method described by Liang et al. (2017). The gene knockout cassette was constructed using three fragments: HygR cassette and the 5′ and 3′ flanking sequences of the target gene (**Table S2**). Then, the individual cassette was introduced into fungal cells using protoplast-mediated transformation. The fungal transformant genomes were obtained following the steps of the cetyltrimethylammonium bromide (CTAB) method (Niu et al., 2008). Correct gene mutants were confirmed by PCR and Southern blot.

### Comparison of Mycelia Growth and Conidiation

Seven- to 10-day-old WT strain and mutant strains were phenotypically analyzed in this study. For the comparison of colony growth, 8-mm diameter hyphal discs were transferred onto the center of 9-cm PDA, TG, and TYGA plates and 6 cm WA plates. After incubating for 7 days, the hyphal diameter of each colony was measured. For the comparison of conidiation, 14-day-old fungal colonies on CMY media were washed with 20 ml ddH_2_O, and conidia yields and their morphologies were next determined through microscope observation. About 100 conidia from the WT strain and mutants were analyzed for germination rate, morphology, and nuclear numbers (Ma et al., 2020). Conidia were spread on WA plates and placed at 28°C. After 12 and 36 h, the germinated conidia were counted.

We used a grinding wheel to divide the cover glass into four parts for sterilization, and then inserted it into a PDA medium containing hyphal disc at 45° for the hyphal-crawling growth. After incubating for 5 days at 28°C, we used the CFW dye with KOH for staining and observation. For conidial staining, we used 5 µl of conidial suspensions for CFW staining and imaging. The hyphal and conidial samples were stained in DAPI dye for 30 min in the dark for nuclear analysis. Thereafter, we used the CFW dye (with 250-fold dilution) for restaining. The images were captured for counting the number of nuclei (Ma et al., 2020).

### Trap Induction and Pathogenicity Analysis

The conidial suspension from 14-day-old WT strain and mutants on CMY medium was washed from the plates. About 1 × 10^5^ conidia were spread over 6 cm of WA plates and cultured for 2 days at 28°C. Thereafter, about 400 nematodes were transferred to the ready mycelia plates. After 6 h at 25°C, we started observing the trap formation and the nematodes captured. The observation lasted for 24 h. For the analysis of ammonia-induction trap formation, we added 1 ml 26.6 µM ammonia solution and observed the trap production for 48 h. The observations were repeated three times.

### Stress Resistance Analysis

The chemicals disturbing the osmosis, oxidation, and cell wall synthesis were used for the stress tolerance tests. We added different doses of sorbitol (0.25, 0.50, and 0.75 M) for osmotic stress, H_2_O_2_ (5, 10, and 15 mM) for oxidative stress, and SDS (0.01%, 0.02%, and 0.03%) for the cell wall perturbing agent into solid TG plates. Then, 8-mm fungal discs from individual mutants were placed on the center of the stress testing plate. After 7 days at 28°C, we observed the fungal morphology and measured the diameters of each plate (Xie et al., 2021).

### pH Tolerance Test

To avoid the effect of high temperature on pH values, we first prepared sterilized TG broth, and then an equal volume of 2.5% agar media was added for the final making of TG plates (pH = 5, 7, and 9). Fungal discs (8 mm) were transferred to the center of the plates. After 7 days, we observed and measured the size of fungal colonies.

### Analyses of Trap Formation and Pathogenicity

Conidia (about 1 × 10^5^) from WT and mutant strains were spread over WA for incubation at 28°C for 36 h. Approximately 500 *C. elegans* or 1 ml of 22.6 µM ammonia solution was added on a ready mycelia plate for the induction of trap formation in *A. oligospora*. Trap formation was observed and counted after 6 h of induction. The captured nematodes were counted under a light microscope at 24 h.

### RT-PCR Analysis

The WT conidial suspension was incubated in CM broth for 48 h at 28°C, and the liquid media was pipetted. The mycelium was exposed to 5 ml of ammonia solution for 2 h. Total RNA from the mycelia was extracted using the TaKaRa RNA extraction kit (Takara, Dalian, China, TaKaRa Bio). qRT-PCR analysis was performed using the TaKaRa kit (PrimeScript™ RT reagent Kit with gDNA Eraser; TB Green™ Premix Ex Taq™ II). Specific paired primers of targeted genes and the internal conference genes were designed using the online software of Primer 3.0 plus (https://bioinfo.ut.ee/primer3-0.4.0/). Transcription levels of sporulation and anti-stress genes were also analyzed. The relative transcription levels (RTLs) of the candidate genes were calculated as the ratio of transcripts in the mutants to that in the WT strain at a given time using the 2^ΔΔCt^ method.

### Statistical Analyses

GraphPad Prism version 8.00 (GraphPad Software, San Diego, CA, USA) was used for the images and the statistical analyses. One-way analysis of variance (ANOVA) followed by Tukey’s multiple comparison test was used to analyze the observations, measurements, and estimates. *p <*0.05 was considered significant. Every experiment was repeated three times.

## Results

### *A rthrobotrys oligospora* Is Abundant in Arrestin Proteins

**Figure 1:**
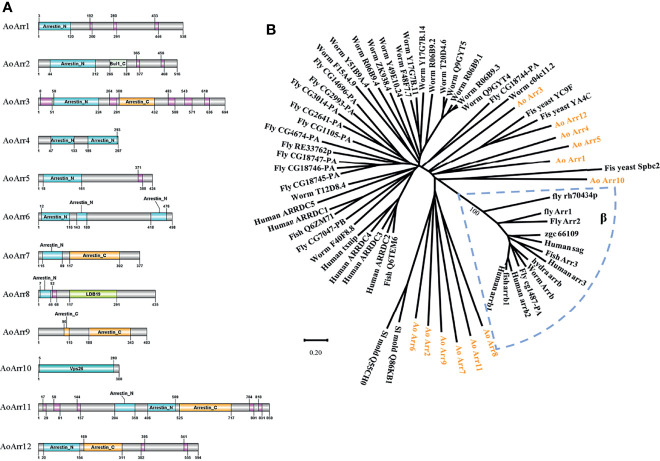
Bioinformatics analysis of arrestin-related proteins. **(A)** Prediction of the domain of arrestin-related proteins in *Arthrobotrys oligospora*. The number represents the number of amino acids in each protein, and the pink module represents low complexity. Among them, Bul1_C, LDB19, and Vps26 domains belong to the arrestin family. **(B)** The phylogenetic tree analysis of arrestin of related species. The 12 arrestin proteins of *A. oligospora* studied in this paper are represented by orange characters, the blue dashed box represents the β-arrestin family, and the remaining black lines represent the α-type arrestin family. The evolutionary tree is drawn using the MEGA6 software.

To determine the abundance of arrestin proteins in the fungus *A. oligospora*, we first retrieved all arrestin proteins from https://www.uniprot.org/uniprot/?. There are 12 members containing highly conserved N- and C-terminal domains in *A. oligospora* (**Figure 1A**). We named these proteins as AoArr1 to AoArr12 (**Table S1**), and the corresponding coding genes were named *AoArc1* to *AoArc12*. The lengths of AoArr proteins are of 297 to 859 amino acids with predicted p*I* from 6.47 to 9.77 and without signal peptides (http://www.cbs.dtu.dk/services/SignalP/). Next, to define whether these AoArrs are type α or type β, we performed phylogenetic tree analysis using AoArrs against others from model organisms of yeast, worm, fly, fish, and human. The phylogenetic tree analysis indicated that all arrestins from *A. oligospora* are completely separated from β-arrestin, indicating that they belonged to α-arrestins (**Figure 1B**). This analysis result is highly consistent with the data obtained in other fungi (Alvarez, 2008; Becuwe et al., 2012; Dong et al., 2016; Telzrow et al., 2019).

### *AoArc* Genes Respond to Ammonia Induction for Trap Formation

**Figure 2:**
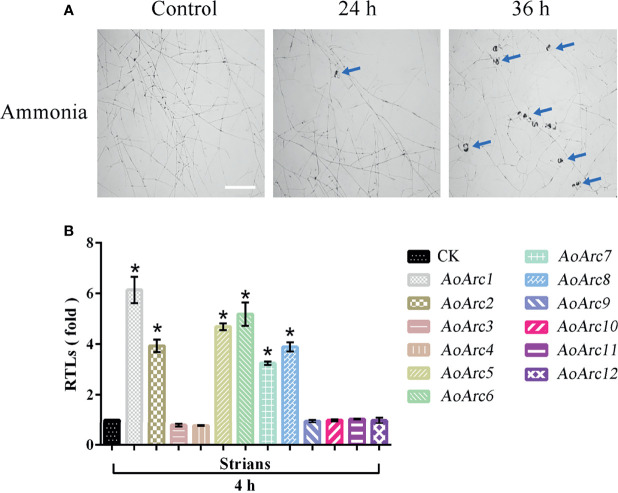
Ammonia-induced expression analysis of *AoArc* genes in *Arthrobotrys oligospora*. **(A)** Ammonia can induce *A. oligospora* to produce traps. With the extension of the ammonia incubation time, the number of traps produced increased significantly, and the blue arrow represents the trap (bar = 500 μm). **(B)** Analysis of the expression of arrestin-related genes after 4 h of ammonia induction. Among them, the samples were cultured at 28°C for 4 h, that is, the arrestin expression of the samples without ammonia induction as a standard (CK), which was used for the statistical analysis of RTL. Each experiment was performed three times. Error bars: standard deviation; asterisk: significant difference between ammonia treatment and no treatment (Tukey’s HSD, *p* < 0.05).

Although nematodes and their extracts can promote trap production in predatory fungi, ammonia had been defined as a simple and effective signaling molecule for the formation of an adhesive network in *A. oligospora* after adding ammonia for 24 h (**Figure 2A**). We analyzed the transcription levels of *AoArc* genes using the transcriptome sequencing data of mycelia samples treated with ammonia for 4 h. Differential analysis results showed that the values of log_2_ fold change in the mycelia after ammonia addition ranged from 0.19668 to 5.6401 (**Table S1**). We then used qRT-PCR to measure the expression difference of ammonia-treated mycelia at 4 h. The expression levels of *AoArc1*, *AoArc2*, *AoArc5*, *AoArc6*, *AoArc7*, and *AoArc8* were increased by over 3-fold (**Figure 2B**). Therefore, we hypothesized that the *AoArc* genes might be involved in the process of trap formation in *A. oligospora*.

### *AoArc* Genes Do not Obviously Affect Fungal Growth in *Arthrobotrys oligospora*

**Figure 3:**
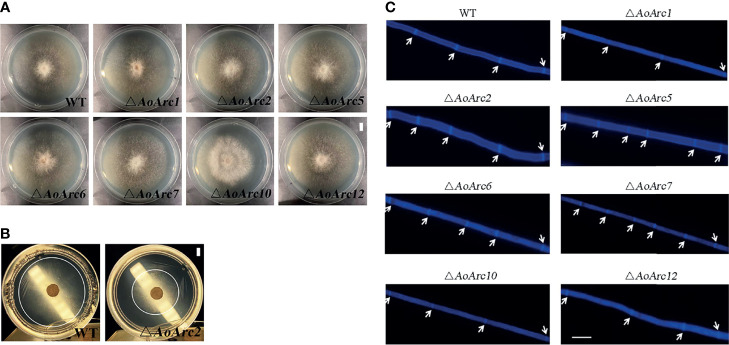
Comparison of hyphae growth and hyphae cell length between WT and *AoArc* mutants. **(A)** Comparison of mycelial growth between wild-type (WT) and *AoArc* mutants; fungal strains were cultured on PDA at 28°C for 7 days (bar = 1 cm). **(B)** Comparison of mycelial growth between WT and *ΔAoArc2* mutant; fungal strains were cultured on WA at 28°C for 7 days. WA is a nutrient-poor medium with weak hyphae growth. The white circle is used to indicate the size of the colony (bar = 1 cm). **(C)** Comparison of hyphal septum between WT and mutants. The white arrow indicates the septum (bar = 20 μm).

To characterize the function of *AoArc* genes, we carried out knockout experiments of the seven *AoArc* genes (*AoArc1*, *AoArc2*, *AoArc5*, *AoArc6*, *AoArc7*, *AoArc10*, and *AoArc12*), of which five were upregulated genes and two were without change after ammonia induction. The corresponding mutants were verified using PCR amplification, Southern blot, and transcriptional level assays (**Figure S1**). Subsequently, all strains were inoculated on the nutrient-rich media of PDA, TG, and TYGA and on the nutrient-poor media of WA at 28°C. The disruption of *AoArc* genes did not inhibit the growth rate on PDA and TG; however, growth was significantly inhibited for Δ*AoArc1* and Δ*AoArc2* on TYGA plates and Δ*AoArc2* on WA plates. The growth of Δ*AoArc2* mutant was nearly 50% and 30% slower than the WT on TYGA and WA plates, respectively, and Δ*AoArc1* on TYGA plates was nearly 30% slower than the WT (**Figures 3A, B** **; Supplementary Figure S2**). Moreover, CFW dye staining demonstrated that the distance between two septa in Δ*AoArc5* and Δ*AoArc7* was shorter than that of the WT (**Figure 3C**). These observations suggested that individual *AoArc* genes, especially *AoArc*1, *AoArc*2, *AoArc*5, *AoArc*6, and *AoArc*7, did not primarily regulate the growth of *A. oligospora* and partially affected the development of hyphae cell length.

### *AoArc* Genes Contribute to Trap Formation and Pathogenicity in *Arthrobotrys oligospora*

**Figure 4:**
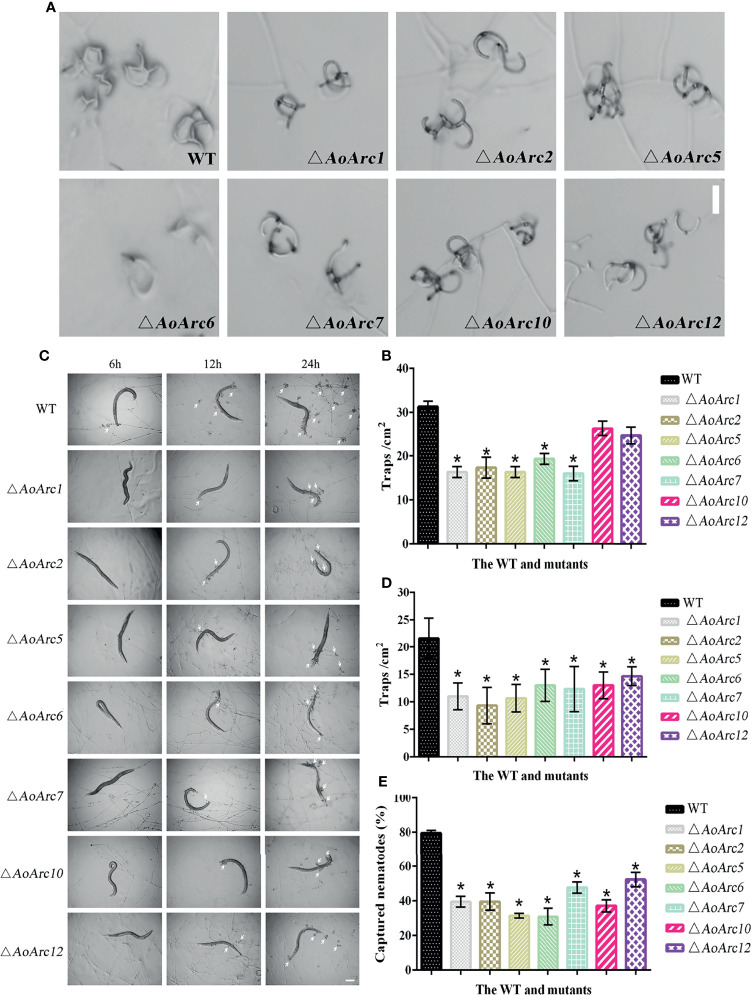
Comparison of trap formation and nematocidal activity between the WT and mutants. **(A)** The formation of traps after 48 h of ammonia treatment (bar = 50 μm). **(B)** Statistics of the number of traps after 48 h of ammonia treatment. **(C)** Trap formation of fungal strains induced by nematodes at 6, 12, and 24 h. White arrows: traps. Bar: 50 μm. **(D)** The number of traps in the WT and mutant strains was compared at 24 h after nematode induction. **(E)** Comparison of captured nematodes by the WT and mutant strains at 24 h. The asterisk indicates a significant difference compared with the WT (*p* < 0.05).

Since the transcriptional levels of *AoArc* genes were changed significantly during ammonia-induced trap formation, we tested the ability of trap formation in *AoArc* knockout strains. The mycelia of all strains could form adhesive nets after ammonia addition for 48 h. However, the level of trap formation in *AoArc* mutant strains of Δ*AoArc1*, Δ*AoArc2*, Δ*AoArc5*, Δ*AoArc6*, and Δ*AoArc7* was significantly lower than that of the WT strain, with 52.12%, 55.31%, 52.12%, 61.70%, and 51.07% of the trap yield compared with the WT strain, respectively. The mutants of Δ*AoArc10* and Δ*AoArc12* were slightly lower than the WT strain (**Figures 4A, B** **)**. For nematode-induced trap formation, *A. oligospora* showed a similar response against ammonia solution (**Figures 4C, D** **)**. Contrary to WT, all *AoArc* mutants could produce adhesive nets and capture nematodes, but the time of trap appearance lagged in all of the *AoArc* mutants. WT strains started to form traps at 6 h after nematode addition, whereas the *AoArc* mutants required 12 h; accordingly, the nematode mortality in all mutants was significantly decreased at 12 h. Prominently, the capture rates of nematodes from Δ*AoArc1*, Δ*AoArc2*, Δ*AoArc5*, Δ*AoArc6*, Δ*AoArc7*, Δ*AoArc10*, and Δ*AoArc12* were 39.44%, 39.56%, 31.38%, 30.87%, 47.71%, 37.09%, and 52.32%, respectively, and that of the WT is 79.51% (**Figure 4E**).

### *AoArc* Genes Contribute to Conidial Morphology

**Figure 5:**
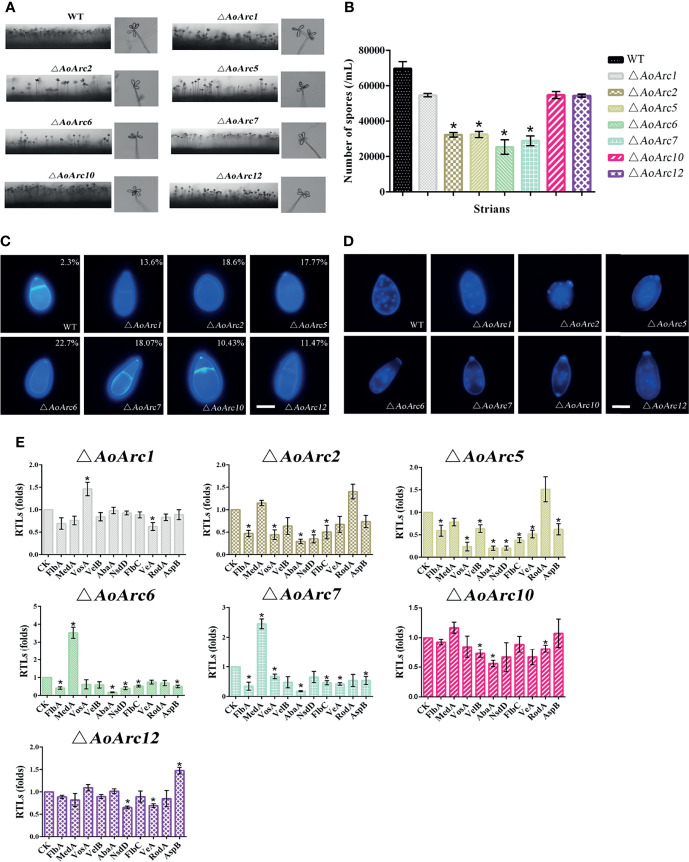
Comparison of aerial hyphae, conidia yield, morphology, number of conidia nuclei, and sporulation-related gene transcription between WT and *AoArc* mutants. **(A)** Comparison of conidiophores between WT and *AoArc* mutants on TYGA at 28°C for 7 days. **(B)** The conidia yields collected from strains grown on CMY for 7 days. **(C)** The spores are stained by CFW, and the percentage in the upper right corner indicates the conidia deformation rate. Bar: 10 μm. **(D)** After the fungal strains were grown on CMY medium for 7 days, the conidia of WT and each mutant strain were collected and stained with 4′,6-diamidino-2-phenylindole (DAPI); the samples were inspected with an inverted fluorescence microscope. Bar: 10 μm. **(E)** Comparison of sporulation-related genes between WT and *AoArc* mutants. CK (*A. o β-tubulin*) is expressed as the standard of RTL statistical analysis. Each experiment was performed three times. Error bars: standard deviation; asterisk: significant difference between mutant and WT (Tukey’s HSD, *p* < 0.05).

To further investigate the effect of *AoArc* genes on conidiophores, mutant and WT strains were grown on TYGA medium for 7 days. In comparison with the WT strain, the number of conidiophores from Δ*AoArc2*, Δ*AoArc5*, Δ*AoArc6*, and Δ*AoArc7* strains on TYGA media was less, whereas the number of conidiophores from Δ*AoArc1*, Δ*AoArc10* and Δ*AoArc12* mutants did not change obviously compared with the WT (**Figure 5A**). Then, the conidia were washed with 20 ml ddH_2_O, and the conidial number was counted under a microscope. There was no significant difference in the conidial yield in Δ*AoArc1*, Δ*AoArc10*, and Δ*AoArc12* strains compared with the WT which produced nearly 7.2 × 10^4^/ml of conidia. On the other hand, the mutants Δ*AoArc2*, Δ*AoArc5*, Δ*AoArc6*, and Δ*AoArc7* had 46.56%, 47.33%, 36.43%, and 41.50% of conidial number compared with the WT strains, respectively (**Figure 5B**). Furthermore, conidial morphology in all strains was analyzed using CFW staining. The deletions noticeably changed the conidial morphology relative to the WT (**Figure 5C**). For example, WT conidia are obovoid and contain one septum near the base of the spores; however, there were relatively high abnormalities (10.43%–22.7%) observed in all *AoArc* mutant conidia. For abnormal conidia, a lack of septum, a change in cell shape, and mislocalized septa were observed. WT conidial morphology also had abnormalities with a low probability of 2.3%.

Additionally, nuclei morphological changes between the WT and mutants were observed after CFW and DAPI staining (**Figure 5D**). The average number of nuclei in a single spore of the WT strain was 13 to 25. However, significant variations in the nuclei distribution were present in all *AoArc* mutants. The number of nuclei reduced to 4 to 12 in each mutant conidium. Similarly, mutants also contained only 4–13 nuclei in uninucleate hyphal compartments, but under the same condition, the WT hyphal nuclei number reached up to 20–23 (**Figures S3A, B**). Meanwhile, the rates of conidial germination in *AoArc2*, *AoArc5*, *AoArc6*, and *AoArc7* strains were reduced nearly by 20% compared with that in the WT strain (**Figure S3C**). Moreover, the expression pattern of sporulation-regulated genes (*FlbA*, *VosA*, *VelB*, *AbaA*, *NsdD*, *FlbC*, *VeA*, *MedA*, *RodA*, and *AspB*) was determined based on the qRT-PCR analysis. These genes were markedly downregulated following the disruption of *AoArc2*, *AoArc5*, *AoArc6*, and *AoArc7* (**Figure 5E**). All the above data demonstrated that *AoArc* genes regulate the conidial development and morphology in *A. oligospora*.

### *AoArc* Genes Contribute to the Endocytosis Process

**Figure 6:**
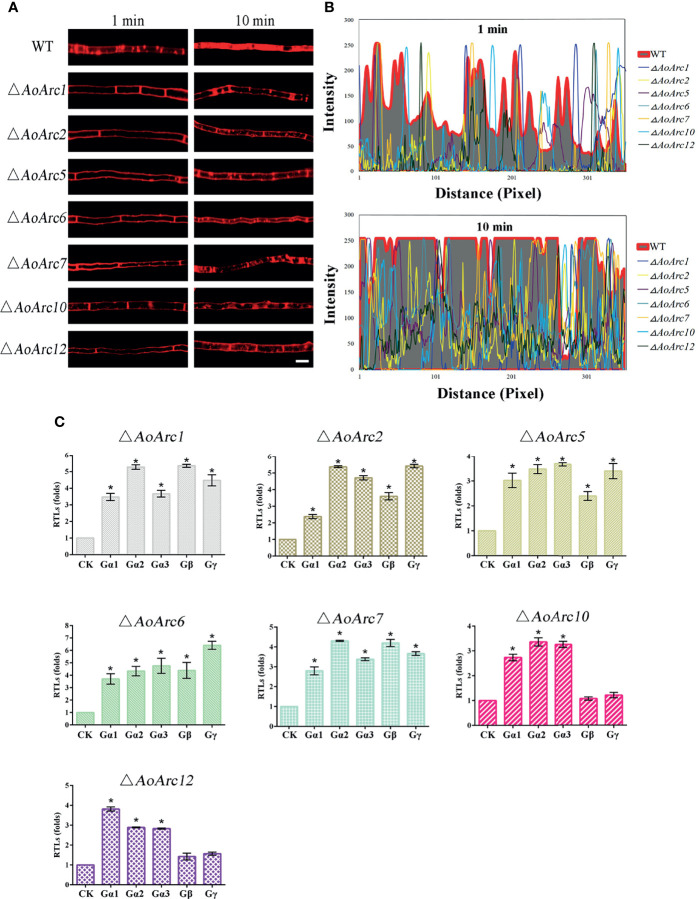
Arrestin is involved in the regulation of fungal endocytosis. **(A)** Comparison of FM4-64 staining between WT and *AoArc* mutants; the samples were inspected with an inverted fluorescence microscope. Bar: 10 μm. **(B)** Analyze the fluorescence intensity of FM4-64 after staining. Top: dyeing for 1 min; Bottom: dyeing for 10 min. **(C)** Comparison of the transcription level of G protein subunits between WT and *AoArc* mutant strains. CK (*A. o β-tubulin*) is expressed as the standard of RTL statistical analysis. Each experiment was performed three times. Error bars: standard deviation; asterisk: significant difference between mutant and WT (Tukey’s HSD, *p* < 0.05).

As lipophilic FM4-64 is an endocytic tracer, we tested the plasma membrane response against the FM4-64 dye for 1 and 10 min. Upon the addition of FM4-64, the plasma membrane was labeled immediately in WT hyphal cells at 1 min. At 1 min, some red fluorescent signals in the WT hyphal cells moved to the cytoplasm from the cell surface, whereas almost none of the dye was confined in the cytoplasm of all *AoArc* mutants under similar conditions. Besides, at exposure for 10 min, large amounts of the dye were retained in the cytoplasm of WT cells, whereas only a few visible dye patches accumulated in the cytoplasm of the mutants. These observations indicate that the deletion of *AoArcs* could block the endocytosis process (**Figures 6A, B** **)**. Thereafter, to confirm whether *AoArc* deletion-initiated endocytosis weakening would affect G-protein signaling, we checked the transcription levels of the five subunits of G protein in this pathway (**Figure 6C**). In Δ*AoArc1*, Δ*AoArc2*, Δ*AoArc5*, Δ*AoArc6*, and Δ*AoArc7* mutants, the RTLs of these subunit genes were significantly upregulated in comparison with the WT strain. In Δ*AoArc10* and Δ*AoArc12* mutants, the expression of genes encoding the three G-protein subunits (*Gα1*, *Gα2*, *Gα3*) was increased by over 2.5-fold, but the genes *Gβ* and *Gγ* were unaffected in the Δ*AoArc10* and Δ*AoArc12* mutants. These data suggested that disruption of arrestin led to activation of G-protein signaling.

### *AoArc* Genes Regulated Stress Resistance in *Arthrobotrys oligospora*

**Figure 7:**
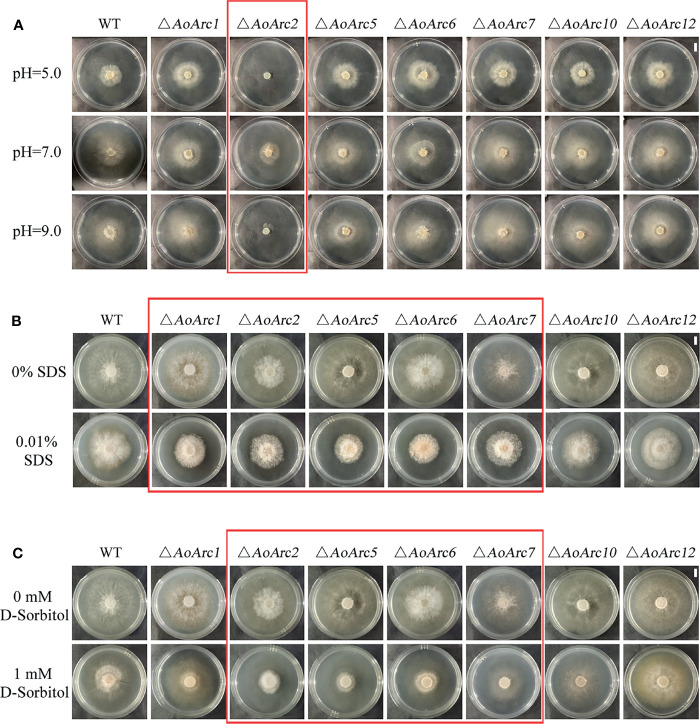
Arrestin responds to stress stimulation. **(A)** Under different pH conditions, comparison of the growth of WT and mutant strains on TG medium. **(B)** Colonial morphology of fungal strains under cell wall-interfering agent stress. **(C)** Colonial morphology of fungal strains under osmotic stress. Bar: 1 cm. Red box: strains with obvious difference effects.

Current models assume that PalF, an α-arrestin protein member, is involved in pH signaling pathway. We examined whether colony growth was different between the *AoArc* mutants and WT strain under acidic (pH 5.0), neutral (pH 7.0), and alkaline conditions (pH 9.0) (**Figure 7A**). The *A. oligospora* WT strain could grow in a wide pH range from 5.0 to 9.0, but only the Δ*AoArc2* mutant strain failed to adapt to the pH variations; other *AoArc* mutants grew under different pH conditions. These data highlighted that among these *AoArc* genes, the *AoArc2* was the dominant pH-response regulator protein in *A. oligospora*.

The ability to resist stressors in the WT and mutants was evaluated using different chemicals: cell wall perturbing agent (SDS), oxidative agent (H_2_O_2_), and osmotic salts (D-sorbitol). We chose the gradient doses of these chemicals for the stress sensitivity tests. Although 0.01% of SDS had a similar inhibitory effect on the growth of WT and mutants of Δ*AoArc10* and Δ*AoArc12*, the effect became more severe in Δ*AoArc1*, Δ*AoArc2*, Δ*AoArc5*, Δ*AoArc6*, and Δ*AoArc7* strains (**Figure 7B**). With respect to D-sorbitol, the mutant strains, especially the Δ*AoArc2*, Δ*AoArc5*, Δ*AoArc6*, and Δ*AoArc7* strains, showed noticeable sensitivity to 1 mM D-sorbitol (**Figure 7C**). *AoArc* mutants displayed higher resistance to 10 mM H_2_O_2_, in which the colony growth of WT was almost completely suppressed, but under similar conditions, the growth of *AoArc* mutants was only nearly 30% (**Figure S4**). Overall, the *A. oligospora* arrestin family played important roles in cellular adaption to external stress and resisting unfavorable conditions.

## Discussion

**Figure 8:**
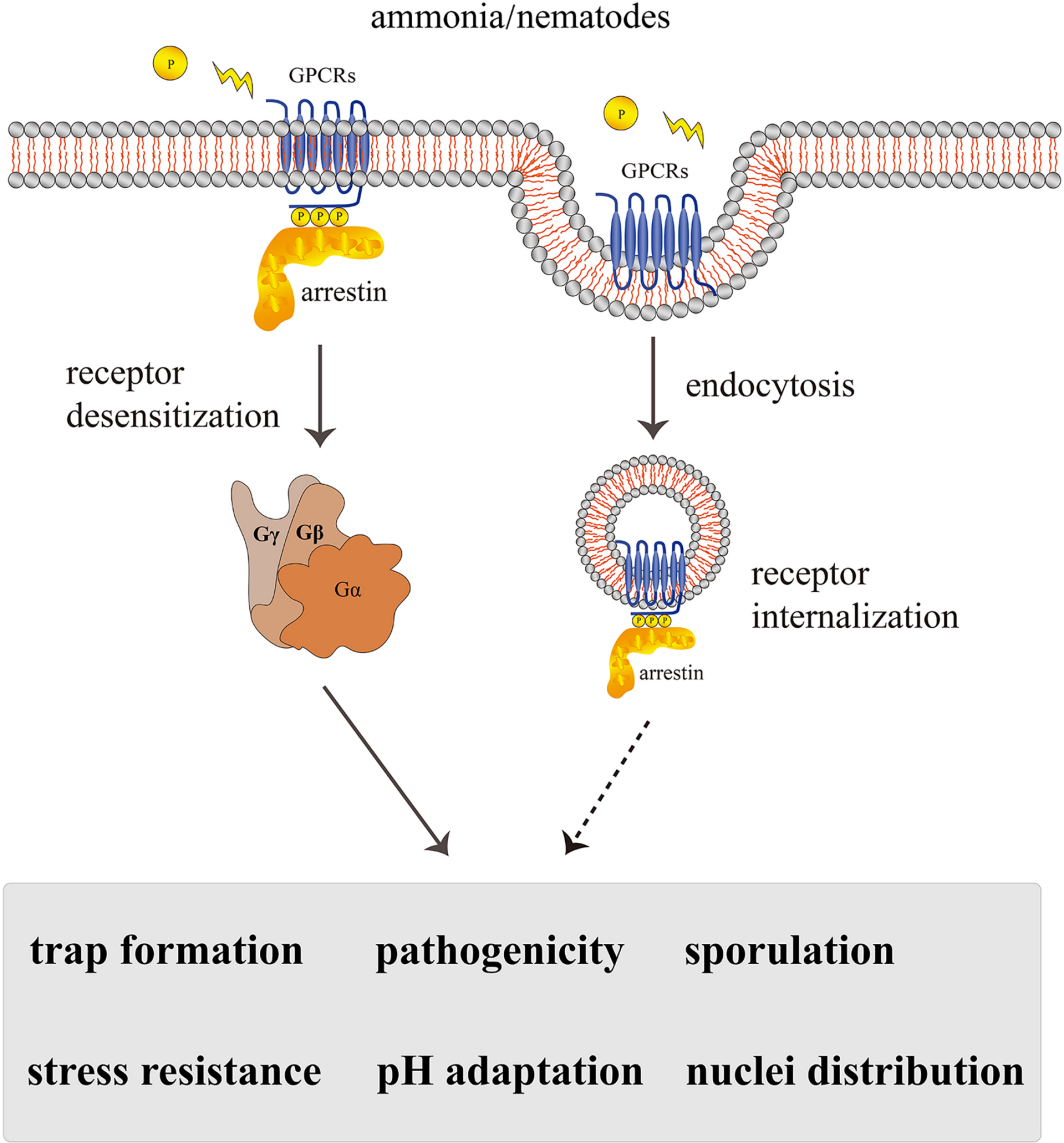
A proposed model for the regulation of *AoArc* genes in *Arthrobotrys oligospora*. *Arthrobotrys oligospora* senses the nematode or ammonia stress signals and transmits them to G protein-coupled receptors in cells. Arrestin triggers various regulatory pathways by inactivating GPCRs and participating in endocytosis, thereby terminating G protein signals to regulate the series of biological processes including trap formation, pathogenicity, sporulation, nuclei distribution, pH adaptation, and stress resistance. The dashed arrow indicates the assumed connection; the solid arrow indicates the experimental result.

Arrestin proteins are abundant in eukaryotes and are important players in terminating the signaling of seven-transmembrane receptors. Accumulating research supports that arrestins act as an adaptor or scaffold by binding PM receptors and subsequently inhibit downstream G-protein signal transduction. Our main results about the functions of arrestins in *A. oligospora* are consistent with the findings in other model organisms. *Arthrobotrys oligospora* arrestins are multifunctional proteins in the regulation of endocytosis, trap formation, pathogenicity, sporulation, nuclei distribution, pH adaptation, and stress resistance. From the classical arrestin signaling paradigm, we suggest that arrestin-regulated multiple cellular morphologies and virulence in *A. oligospora* may be directly linked to the receptor endocytosis pathway (**Figure 8**).

The NTF *A. oligospora* has 12 arrestin members. The higher abundance of arrestins suggests that these proteins are important for the regulation of cellular processes. Based on structure, arrestin can be classified as visual and β-arrestins, α-arrestins, and Vps26-related proteins, and these subgroups have similar regulatory functions in different organisms (Kang et al., 2014). In mammals, visual and β-arrestin-mediated endocytosis has been widely proposed in GPCRs and other receptor desensitization (Beautrait et al., 2017). For instance, disruption of β-arrestins can block the glucocorticoid receptor, severely retarding lung and liver development in mice (Zhang et al., 2011). In yeast, 13 α-arrestins mediate endocytosis and PM quality control *via* Rsp5 ubiquitin ligase–ART adaptor networks (Nikko and Pelham, 2009). The lack of Rsp5 adaptors in yeast cells is likely to cause the accumulation of PM proteins on the cell surface, causing proteotoxic stress (Zhao et al., 2013). Although we did not demonstrate the interaction between AoRsp5 (AOL_s00188g58) and AoArr in this study, we confirmed that most of the AoArr proteins contain PxY motifs required for binding Rsp5 in yeast and animal cells (**Table S1**). Therefore, the regulatory role of AoArrs in *A. oligospora* may be similar in yeast and other eukaryotes.

Importantly, lifestyle conversion in predatory fungi is crucial for the fungal infection process. A previous study from our team has shown that G proteins and MAPK are important for signal transduction mechanism in fungi, and mutations of the MAPK protein AoSlt2, AoIme2, and Rab protein AoRab-7A can affect hyphal growth, cell nucleus development, conidiation, trap formation, and pathogenicity in *A. oligospora* (Yang et al., 2018; Zhen et al., 2018; Xie et al., 2020). Besides, G-protein β-subunit gpb1 mutants were significantly defective in trap morphogenesis in response to *C. elegans* (Yang et al., 2020). As arrestins block the signal transduction by preventing the interaction between cytoplasmic receptor domains and heterotrimeric G proteins (Herranz et al., 2005), we hypothesized that the loss of arrestins would increase GPCR retention on the PM, blocking the endocytosis process. Our observations using FM4-64 dye staining supported this speculation. Noticeably, FM4-64 internalization was significantly weakened in all *AoArc* mutants. Meanwhile, arrestin gene disruptions also lead to lower levels of the trapping cell formation and nematode deaths. Although inhibition of endocytosis was demonstrated in this study, its physiological importance in the NTF remains largely unaddressed. It seems that endocytosis is linked to fungal pathogenicity, as arrestin mutants demonstrated reduced trap formation and capturing of nematodes. The role of G proteins in regulating trap formation and pathogenicity in NTF is very well established. Thus, it is possible that as scaffolds associated with GPCRs, arrestin proteins are involved in controlling NTF growth pattern conversion and pathogenicity by regulating endocytosis. Therefore, interruption of various proteins in the G-protein signaling pathway may affect the normal growth and development of traps in *A. oligospora*.

Moreover, the importance of the Pal/Rim pathway for fungal adaptation to ambient pH has been proven in *Beauveria bassiana*, *Aspergillus nidulans*, and *Saccharomyces cerevisiae*. This pathway involves seven proteins: PalH, PalI, PalF, PalC, PalA, PalB, and PacC (Selvig and Alspaugh, 2011; Zhu et al., 2016). After sensing the extracellular alkaline pH, membrane proteins PalH and PalI providing pH-sensing activity are bound by arrestin-related protein PalF. PalF is phosphorylated and ubiquitinated in a PalH-dependent manner, indicating PalF as the possible link between ambient pH sensor(s) (Landraud et al., 2013; Zhu et al., 2016). These cascades mediate fungal adaptation to environmental pH. Our results displayed that *AoArc2* deletion caused severe growth defects in mutants compared with the WT strain under acidic and alkaline conditions, which is consistent with the observation in PalH mutant, the most upstream of all other Pal proteins, causing similar growth defects: *A. oligospora* lacking *AopalH* has reduced conidiation and formation of traps. Thus, arrestins similarly regulate pH adaptation in *A. oligospora*.

Furthermore, α-arrestins regulate conidiation patterns in *Magnaporthe oryzae*. *ARRDC1* (Arrestin domain containing 1) is considered as an autophagy-associated protein. The absence of *ARRDC1* affects the expression profile of virulence-related transcription factor CCA1 and generates an acropetal array of conidia (Banguela-Castillo et al., 2020). After the co-localization of ARRDC1 and Autophagy-related protein 8 (ATG8), other arrestin proteins enter the autophagic flux before autophagosome maturation (Dong et al., 2016). Our results showed that mutations in arrestin-coding genes result in a high proportion of defective conidia, and the nuclei numbers in each hyphal compartment and conidial cells are almost halved compared with the WT cells. This observation is relative to the autophagy regulation pathway in which cellular components, including nuclei, are degraded (Corral-Ramos et al., 2015). ATG5 mutation in *A. oligospora* caused a significant reduction in the number of cell nuclei, as found in the case of arrestin mutations (Zhou et al., 2021). Therefore, arrestin protein is an important factor in regulating conidial development in *A. oligospora*.

Finally, for responding to these unfavorable conditions, mammalian and model fungal arrestins help regulate viability and virulence. Our data showed that arrestins are important for the adaption of *A. oligospora* to several stress conditions. In the analysis of oxidative, hyperosmotic, and cell wall perturbing stresses, mutants revealed increased sensitivity toward stress cues. Furthermore, the stress-regulated genes were also affected. In animal cells, the knockdown of β-arrestin has been shown to decrease ROS and NADPH oxidase 4 (NOX4) expressions (Sun et al., 2020). The α-arrestin protein Ali1 in *Cryptococcus neoformans* acts as a novel regulator of cytokinesis in the presence of stress. In the *ali1* mutant, cell surface integrity was impaired in the presence of various cell surface stressors, such as calcofluor white, Congo red, SDS, and caffeine. Individual arrestin mutants display distinct, but overlapping, phenotypes in the presence of acidic (pH 5.0) and alkaline (pH 9.0) conditions, cell wall perturbing agent (SDS), oxidant agent (H_2_O_2_), and osmotic salts (D-sorbitol) (Telzrow et al., 2019). In addition, in the soil amoeba *Dictyostelium*, the arrestin-domain-containing protein AdcA also helps to respond to a variety of stressors, such as glucose, glycerol or NaCl, heat shock, mitochondrial uncoupler FCCP, and oxidative stress. Therefore, arrestin proteins facilitate the maintenance of cell integrity and cellular functions against various extracellular stressors.

This study demonstrates that 12 arrestin proteins in *A. oligospora* play multifaceted roles in response to environment change. The trap signaling molecule, such as ammonia, triggers the higher expression of six arrestins (*AoArc1*, *AoArc2*, *AoArc5*, *AoArc6*, *AoArc7*, and *AoArc8*) to facilitate fungal lifestyle switch, and cellular differentiation may be mediated by endocytosis. Arrestin-related proteins mediate pH signaling as an important mechanism through which fungi can adapt to a changed pH condition. Similar to yeast *PalF*, the *AoArc2* is the dominant pH-response regulator protein in *A. oligospora*. Through a series of analysis of phenotypes on growth, stress resistance, virulence, and conidiation, *AoArc2*, *AoArc5*, *AoArc6*, and *AoArc7* all regulate these process. Considering the high conservation of arrestin family members, we did not investigate the roles of the remaining five genes (*AoArc3*, *AoArc4*, *AoArc8*, *AoArc9*, and *AoArc11*). Thus, it is highly possible that *AoArc8* has a similar function with *AoArc1*, *AoArc2*, *AoArc5*, *AoArc6*, and *AoArc7*, and *AoArc3*, *AoArc4*, *AoArc9*, and *AoArc11* are similar with those of *AoArc10* and *AoArc12.*

Overall, arrestin proteins serve as the scaffolding molecules linking membrane receptors to multiple cytosolic proteins for initiating multiple signal transduction processes. Through arrestin-bound receptor internalization, cells maintain the dynamics and stability of the plasma membrane in response to external cues. Despite the mechanistic novelty of the fungal arrestin signaling transduction pathway, this work provides robust evidence to delineate the multifaceted functions of arrestin proteins in conidiation, stress adaptation, morphogenesis, trap-related pathogenesis, and endocytosis.

## Conclusion

There are 12 α-arrestin proteins containing the N- and C-arrestin domain in the NTF *A. oligospora.* They are highly functionally and structurally conservative proteins. Here, the role of seven *AoArc* genes was analyzed in the regulation of growth, conidiation, trap formation and pathogenicity, endocytosis process, and pH signaling. Our study provides the possible mechanism of trap formation where arrestin-mediated endocytosis regulates the trap-structure biogenesis in NTF. Besides, further investigation is needed to unveil how arrestin proteins interact for tuning PM stability after the environmental stimuli.

## Data Availability Statement

The original contributions presented in the study are included in the article/**Supplementary Material**. Further inquiries can be directed to the corresponding authors.

## Author Contributions

XW and GL conceived and designed the study. LZ, XW, and GL wrote the manuscript. LZ and ML conducted the experiments. PC, MT, YX, and ML analyzed the data. XW, GL, XZ, and KZ revised the manuscript. All authors contributed to the article and approved the submitted version.

## Funding

This research was jointly supported by the National Natural Science Foundation of China (31760024 and U1802233), the Applied Basic Research Foundation of Yunnan Province (2018FB024, 2019FB123), the Department of Science and Technology of Yunnan Province (202001BB050061), and the Scientific Research Fund Project of Yunnan Provincial Department of Education (2021Y048).

## Conflict of Interest

The authors declare that the research was conducted in the absence of any commercial or financial relationships that could be construed as a potential conflict of interest.

## Publisher’s Note

All claims expressed in this article are solely those of the authors and do not necessarily represent those of their affiliated organizations, or those of the publisher, the editors and the reviewers. Any product that may be evaluated in this article, or claim that may be made by its manufacturer, is not guaranteed or endorsed by the publisher.
