# Supplementary material for: Arrestin-Coding Genes Regulate Endocytosis, Sporulation, Pathogenicity, and Stress Resistance in Arthrobotrys oligospora
Source: Front Cell Infect Microbiol. 2022 Feb 16;12:754333. doi: 10.3389/fcimb.2022.754333 (PMC8890662; doi:10.3389/fcimb.2022.754333)
Supplement: Supplementary file 1 [file DataSheet_1.docx]

**Supplementary materials**

1. **Supplementary Figures**

**
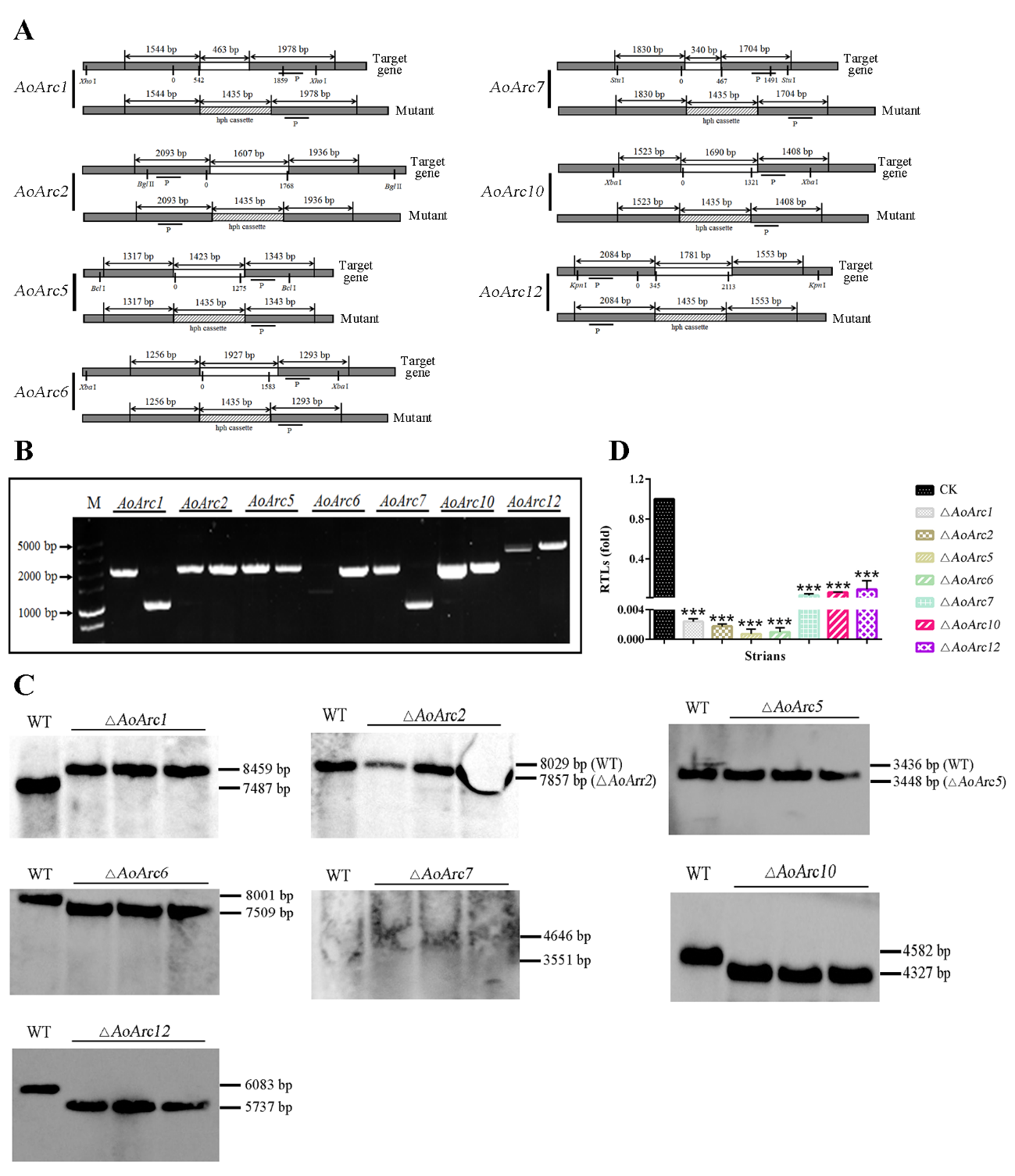
**

**Figure S1.** Knock-out and verification of arrestin coding gene in *A. oligospora*. (A) Diagrammatic sketch of homologous recombination of arrestin. The homologous flanking sequences of the target gene, Southern blot probe (p), and the restriction enzyme sites were marked. (B) Putative transformants were verified by PCR. Among arrestin-related genes, the left lane represents WT, and the right lane represents mutant. (C) The transformant was confirmed by Southern blot. Each mutant was validated with 3 transformants. (D) Analysis of arrestin expression of knockout gene mutants and WT. Among them, all strains were grown on PDA for 5 days, and the arrestin expression level of WT was used as standard (CK) for RTL statistical analysis. Each experiment was performed 3 times. Error bars: standard deviation, asterisk: significant difference between mutant and WT (Tukey's HSD, p<0.05).


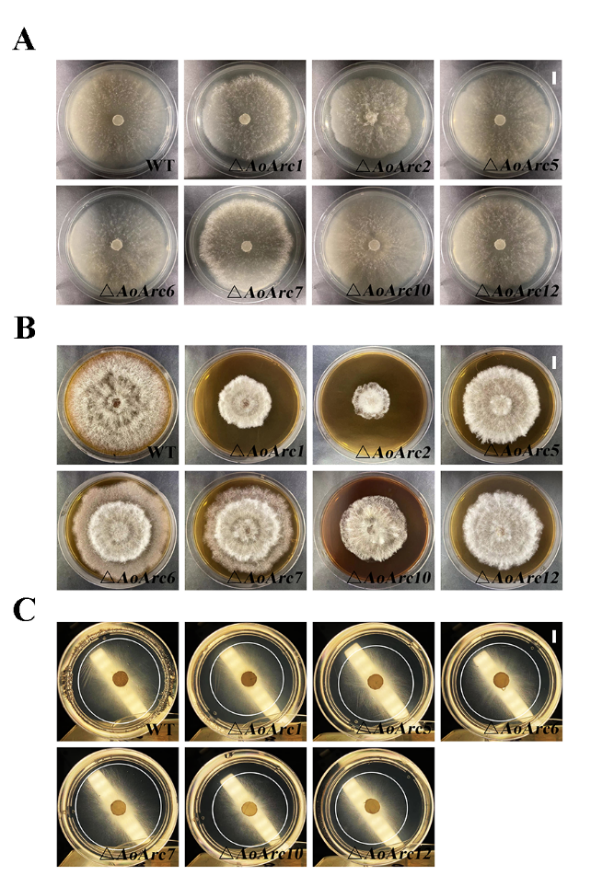


**Supplementary Figure S2.** Comparison of wild-type and mutant strains growing in different media. (A) Comparison of the growth of wild type and mutant strains in PDA. （B）Comparison of the growth of wild type and mutant strains in TYGA.（C）Comparison of the growth of wild type and mutant strains in WA. The comparison between WT and *ΔAoArc2* is shown in Figure 3B. Bar: 1 cm.


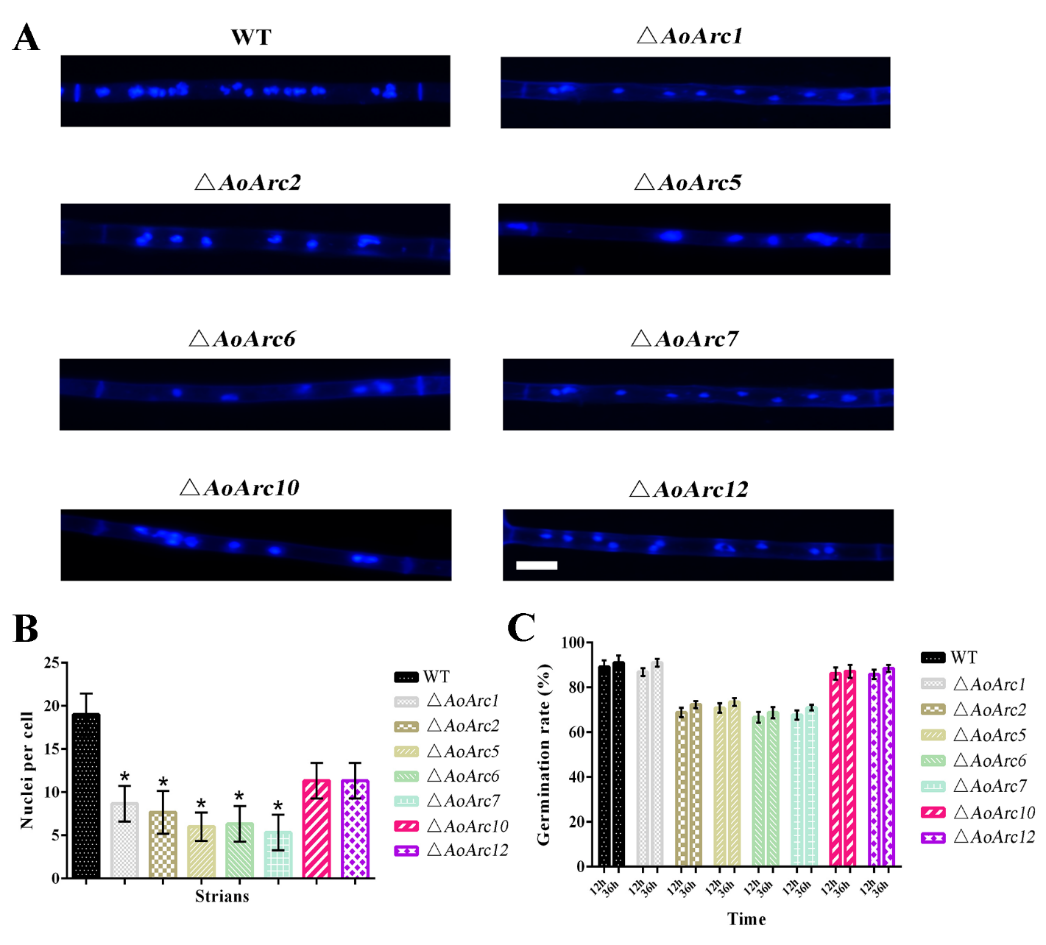


**Supplementary Figure S3.**  Comparison of hyphae nucleus and conidia germination rate of wild-type and mutant hyphae. (A) Comparison of wild-type and mutant mycelial cell nuclei with DAPI staining, the samples were inspected with an inverted fluorescence microscope. Bar: 10 μm. (B) Statistics of the number of nuclei of WT and mutant hyphae. Each experiment was performed 3 times. Error bars: standard deviation, asterisk: significant difference between mutant and WT (Tukey's HSD, p <0.05). (C) Comparison of wild-type and mutant hyphae germination rate.


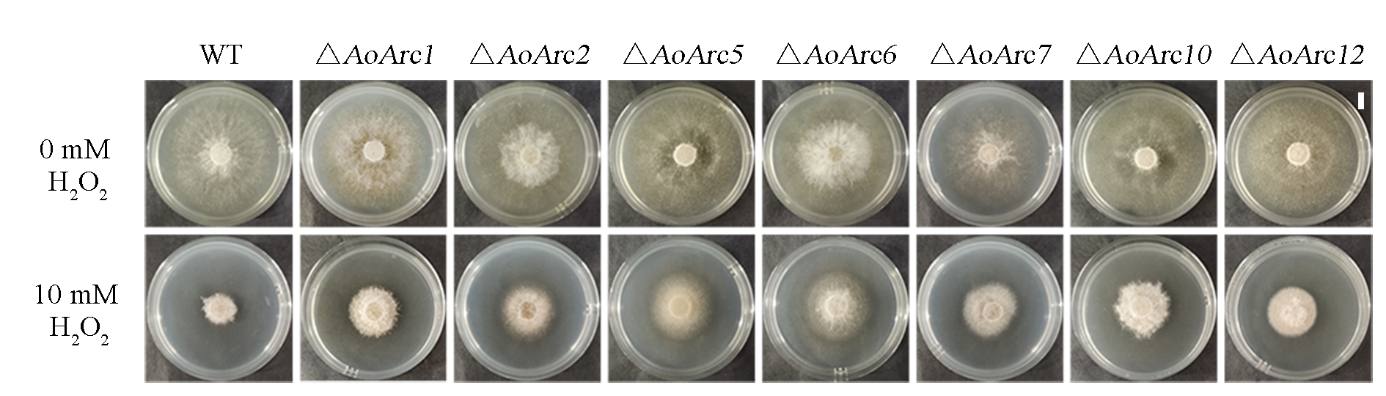


**Supplementary Figure S4**. Comparison of H_2_O_2_ response between the WT and mutants. Bar: 1 cm.

**2. Supplementary tables**

**Table S1. Identification of 12 arrestin proteins.**

| **Gene number** | **protein** | **Number of amino acids** | **pI prediction^*^** | **values of log2Fold change** | **PxY motifs** |
| --- | --- | --- | --- | --- | --- |
| AOL_s00193g25 | AoArr 1 | 538 | 9.26 | 5.4197 | ＋ |
| AOL_s00193g74 | AoArr 2 | 516 | 7.80 | 5.1714 | ＋ |
| AOL_s00076g17 | AoArr 3 | 694 | 7.68 | N | ＋ |
| AOL_s00091g8 | AoArr 4 | 297 | 8.79 | N | ＋ |
| AOL_s00043g250 | AoArr 5 | 424 | 6.47 | 4.3886 | ＋ |
| AOL_s00117g61 | AoArr 6 | 498 | 7.64 | 5.6401 | ＋ |
| AOL_s00054g332 | AoArr 7 | 377 | 9.77 | 3.552 | － |
| AOL_s00215g179 | AoArr 8 | 435 | 6.90 | N | ＋ |
| AOL_s00097g169 | AoArr 9 | 403 | 8.85 | 0.19668 | ＋ |
| AOL_s00076g674 | AoArr 10 | 300 | 6.63 | 0.55389 | ＋ |
| AOL_s00004g452 | AoArr 11 | 859 | 9.00 | 2.4672 | ＋ |
| AOL_s00004g345 | AoArr 12 | 594 | 6.65 | 0.2935 | ＋ |

*: pI is predicted by <https://web.expasy.org/compute_pi/>

N: Transcriptome not detected

+/-: Exist/not exist

**Table S2. List of primers used for gene knockout in this study.**

| **primers** | **Paired sequences** | **Purpose** |
| --- | --- | --- |
| AoArc1-5F | AGCCATTGACGGGAGAAG | Amplify the 5’ fragment |
| AoArc1-5R | GGAAGGCGGAAGAGTTGT |  |
| AoArc1-3F | TATTCTGCGTTCACTCCA | Amplify the 3’ fragment |
| AoArc1-3R | TCAACTTCCAGGCTATTC |  |
| YZ AoArc1-F | AGGAACCTAAGCGGCACG | Verify the transformants |
| YZ AoArc1-R | GAACCGAACTGGGAAATG |  |
| AoArc2-5F | CAGCACGGCTCCGCATAA | Amplify the 5’ fragment |
| AoArc2-5R | CGGGTGGTGAAAGAGTAGGG |  |
| AoArc2-3F | ACACGATAAGCGTCTAAC | Amplify the 3’ fragment |
| AoArc2-3R | AACCCAAATCTTCTCACTA |  |
| YZ AoArc2-F | TAGTATTGGGTGTAGGCTCTT | Verify the transformants |
| YZ AoArc2-R | GGCATGATTATGGGACGT |  |
| AoArc5-5F | TCCTGTAGACTTGCCGACTT | Amplify the 5’ fragment |
| AoArc5-5R | CTGACGGTTTCTTGTGCC |  |
| AoArc5-3F | GGGAGATGTGGTGTTTACG | Amplify the 3’ fragment |
| AoArc5-3R | TACGGTGGACGAGACGAT |  |
| YZ AoArc5-F | GAGTTCCCATTGTAAGCG | Verify the transformants |
| YZ AoArc5-R | CGAGGGCAATACCACTTC |  |
| AoArc6-5F | CAACCCTCCAGCCACTCT | Amplify the 5’ fragment |
| AoArc6-5R | AAGTTACAGGACGGGAAA |  |
| AoArc6-3F | TGATGACCCTATTCTGACG | Amplify the 3’ fragment |
| AoArc6-3R | CCGCATTTCGCTTCTAAC |  |
| YZ AoArc6-F | AAGCATTTGGGTGCGTAT | Verify the transformants |
| YZ AoArc6-R | CTATCTTGCTGGCTTGGC |  |
| AoArc7-5F | ACATAGCCGACGTTCTACTGT | Amplify the 5’ fragment |
| AoArc7-5R | CGCCCTTCCTGATACTCC |  |
| AoArc7-3F | CCTAAGCCGCCCTTACTG | Amplify the 3’ fragment |
| AoArc7-3R | GATGGAGCCACGATCACC |  |
| YZ AoArc7-F | CACATTGCTGGTGGGTCG | Verify the transformants |
| YZ AoArc7-R | TTACGGATGCCAACTTCA |  |
| AoArc10-5F | AGGCGGAGCCAAGATAAT | Amplify the 5’ fragment |
| AoArc10-5R | GCTACAAACCTTACCCTTTC |  |
| AoArc10-3F | TGCGGAGGAGTTCGTGAC | Amplify the 3’ fragment |
| AoArc10-3R | TTGTGAATCGGACTTTGG |  |
| YZ AoArc10-F | GTTCAGACGGTAATAATAGGC | Verify the transformants |
| YZ AoArc10-R | AAGCAATAGGATGCAGGA |  |
| AoArc12-5F | CTCGCACTGTCGCCCTAT | Amplify the 5’ fragment |
| AoArc12-5R | TCCGTGGAAGATTTCATTTA |  |
| AoArc12-3F | TGGCTACACCTTTCACAA | Amplify the 3’ fragment |
| AoArc12-3R | ATCTACCCTCGCTCACCC |  |
| YZ AoArc12-F | TTCTAACGCCATTGCTCG | Verify the transformants |
| YZ AoArc12-R | TGAAGTCGCCTCATCCAC |  |
| Hph-F | GTCGGAGACAGAAGATGATATTGAAGGAGC | Amplify the hph cassette |
| Hph-R | GTTGGAGATTTCAGTAACGTTAAGTGGAT |  |

**Table S3.** Paired primers used for Southern blot analysis of genes associated with arrestin.

| **primers** | **Paired sequences** | **Probe length** |
| --- | --- | --- |
| AoArc1P-F | CAAAGGAAGTGAAGGGGC | 495 bp |
| AoArc1P-R | GCAAGGGAATACCGAAAGA |  |
| AoArc2P-F | GCATCTATCTACACAACCACCA | 412 bp |
| AoArc2P-R | TTTTACTCCACCCCACTCC |  |
| AoArc5P-F | GTTGTAAAAGGACACCCCC | 469 bp |
| AoArc5P-R | AAAAGCCAATTATATTAAGACGG |  |
| AoArc6P-F | TCTATCTTGCTGGCTTGGC | 434 bp |
| AoArc6P-R | GCTTTCGTGATTGGATTTCTTA |  |
| AoArc7P-F | CTCATCGTCGCAAAGTCAA | 599 bp |
| AoArc7P-R | TCCCAACCGCAAGCC |  |
| AoArc10P-F | GCCCCATACTGCTTTGC | 412 bp |
| AoArc10P-R | GACGCCTTCCGGCTAAT |  |
| AoArc12P-F | GCAATGAGGGTGGGTGA | 587 bp |
| AoArc12P-R | CGAGCAATGGCGTTAGAAG |  |

**Table S4.** Paired primers used for RT-PCR analysis of genes associated with arrestin, conidiation and G protein subunits in *A. oligospora*.

| **Arrestin related genes** | **Paired sequences (5'-3')** |
| --- | --- |
| AOL_s00193g25 (*AoArc1*) | GTATTCCGCAAACTGGCATT/ CGCCATATCATGGGGTATTC |
| AOL_s00193g74 (*AoArc2*) | ACCACGAAGCTCAAGTACCG/ GAGTCGTTCTCTGGGTCTGC |
| AOL_s00076g17 (*AoArc3*) | GGCGAAGGACGGTTAGGTGC/ GGTCGGGTAAGGGTAGCAGT |
| AOL_s00091g8 (*AoArc4*) | CGCCTAAAGTTTGAGCTTCG/ GCTCGGCAACCAAGTTACTC |
| AOL_s00043g250 (*AoArc5*) | ACTGCGACCTGGTATGGAAC/ ATACCGTGTGAGTCGGGAAG |
| AOL_s00117g61 (*AoArc6*) | CCGGATCAAGAGTCTCAAGC/ CTCCCAGTTGTGTCCGACTT |
| AOL_s00054g332 (*AoArc7*) | AAGTTGGCATCCGTAAAACG / TGTCGAAAGACGTTGAGGTG |
| AOL_s00215g179 (*AoArc8*) | GCAGTCCACCTCGACCACCG/ TGTGTTTCCTTTACTGGATC |
| AOL_s00097g169 (*AoArc9*) | GCGAATAGAAGGCAAAGTCG/ CAACAAGGTCGGTCCATTCT |
| AOL_s00076g674 (*AoArc10*) | CGGGCATCAAGGTTCAGTTC/ GCTTGACATTTATGCCGTTG |
| AOL_s00004g452 (*AoArc11*) | CAGCGGAGGCCTACTTACTG/ GACCTTGACCTTGGAAGACG |
| AOL_s00004g345 (*AoArc12*) | GCGAAGCGTTTACCTCTCAG/ ACGGCTTTGCATGGTGTAGT |
| **Sporulation genes** | **Paired sequences (5'-3')** |
| AOL_s00169g18 (*VeA*) | AAGCTACACCCAATCAACGC/ TTGCGATGCTGACGATCTTG |
| AOL_s00007g157 (*FlbC*) | CTCTCCGGCAAAGACAATCG/ GTCGACTGAGGATAGTAGCT |
| AOL_s00075g211 (*NsdD*) | ATTACGGCCGCCTAGTAGTC/ CTCGTTTGGACCTGGTTGTG |
| AOL_s00006g570 (RodA) | GCGGATCCAACATGAAGCTT/ GGTTGACAACTGGGATGCTG |
| AOL_s00054g700 (*VosA*) | CAAACCACCCACCACCAAAT/ GGATGGACAGGAGAAGGACC |
| AOL_s00080g63 (*AbaA*) | AACTTTATGCGCCTTGTCGT/ TTGGCTAGGTGGTCTGTACG |
| AOL_s00215g516 (*FlbA*) | TTCAAACGCAGCTCCTTCAC/ AAGCGGGTTGACAGATGAGA |
| AOL_s00210g120 (*MedA*) | TCCGGCCCAATGATTCAGAA/ AGATCGCAGGAACATGGTGA |
| AOL_s00054g811 (*VelB*) | ATTCCGCAACTTCTCCCTCA/ GGCATGTTTGGATTCTGGGG |
| AOL_s00215g893 (*AspB*) | ATACCGCCAACACCCTCTAC/ AACCATCTTCATCTCGGCCT |
| **G protein related genes** | **Sequence (5′-3′)** |
| AOL_s00075g181 (*Gα1*) | TTGCTATTGGGTGCTGGTG/ GATTGAACGGTGTTGGAGAA |
| AOL_s00075g14 (*Gα2*) | AGAGCGGTATCGGGGAATAA/ GGTGCTAGATGGTCCCTTGG |
| AOL_s00109g19 (*Gα3*) | GTGTCGCATTGAGCGAATACGACCA/ GCCTGAACAGATCCACCTTG |
| AOL_s00083g167 (*Gβ*) | CCGTTCACAACCAAGCAGG/ GTCTTTCCAAGTCTTCCCGTAG |
| AOL_s00004g438 (*Gγ*) | AGCACTGACCGCCGACAT/ AGCACTGACCGCCGACAT |
| **β-tubulin gene** | **Sequence (5′-3′)** |
| AOL_s00076g640 | CCACCTTCGTCGGTAACTC/ TCGTCCATACCCTCACCAG |
